# Sauce for the Goose

**Published:** 1885-11

**Authors:** 


### "SAUCE FOR THE GOOSE."

We have always thought, and we cannot but keep thinking, that if sauce for the goose is *not* sauce for the gander, it ought to be. We advocate consistency; and while it is exceedingly disagreeable to have to differ with Dr. N. S. Davis and the Committee, whose cause we have supported to the best of our ability, we must say, that inasmuch as this fight was made on the ground that the Committee, being a creature of the American Medical Association, was responsible to that body; and—inasmuch as we and Dr. Davis, and many others, have claimed and asserted, and truthfully claimed and asserted—that the Committee was not set aside, nor changed, but only *enlarged*,—that the present Committee is not a "New Committee," but the original Committee *enlarged* by the addition of one member from each State—it is as much obligated to report back to the American Medical Association, at its next meeting, as it was at the time Dr. Billings made the report at New Orleans. It has been asserted and reiterated that the Committee was, from the first, and is now, *only* a Committee of Arrangements; and if we were right in demanding that it should so report,—and that its acts were subject to revision by the Association, we can see *no* reason whatever why it should not be required to report at St. Louis,—nor why its acts are not just as much subject to revision as they were in the first place.

The Carthagenian ship had been repaired so often that, finally, there was not a particle of the original ship left; but its identity was not thereby changed—it was the same ship; and it matters not if ALL the original members of this Committee had resigned, so their places were filled,—it is the *same* Committee; and what applied to it at New Orleans certainly should apply to it at St. Louis—it must report and be discharged, and its report may, or may not be received.—We are of opinion however that it will be; –we are only arguing that we do not know of anything having happened to alter the relation of the committee to the source whence it received its power—We have not "heard anything drap."

In this connection, we would remind the *New York Medical Record* that it was *S. W. Gross*, M. D., who appealed to Speaker Randall, and not Dr. Davis. There is some difference between me-um and tu-um, if you will look at it right, and it *does* make a difference whose ox is gored.

